# Noisy Galvanic Vestibular Stimulation Promotes GABA Release in the Substantia Nigra and Improves Locomotion in Hemiparkinsonian Rats

**DOI:** 10.1371/journal.pone.0029308

**Published:** 2012-01-06

**Authors:** Ghazaleh Samoudi, Hans Nissbrandt, Mayank B. Dutia, Filip Bergquist

**Affiliations:** 1 Department of Pharmacology, Institute of Neuroscience and Physiology, The Sahlgrenska Academy, University of Gothenburg, Göteborg, Sweden; 2 Centre for Integrative Physiology, University of Edinburgh, Edinburgh, United Kingdom; 3 Department of Neurology, Sahlgrenska University Hospital, Göteborg, Sweden; Oslo University Hospital, Norway

## Abstract

**Background:**

The vestibular system is connected to spinal, cerebellar and cerebral motor control structures and can be selectively activated with external electrodes. The resulting sensation of disturbed balance can be avoided by using stochastic stimulation patterns. Adding noise to the nervous system sometimes improves function. Small clinical trials suggest that stochastic vestibular stimulation (SVS) may improve symptoms in Parkinson's disease. We have investigated this claim and possible mechanisms using the 6-hydroxydopamine (6-OHDA) hemilesion model of Parkinson's disease.

**Methodology/Principal Findings:**

Animals were tested in the accelerating rod test and the Montoya staircase test of skilled forelimb use. In 6-OHDA hemilesioned animals, SVS improved rod performance by 56±11 s. At group level L-DOPA treatment had no effect, but positive responders improved time on rod by 60±19 s. Skilled forelimb use was not altered by SVS. To investigate how SVS may influence basal ganglia network activity, intracerebral microdialysis was employed in four regions of interest during and after SVS. In presence of the γ-amino buturic acid (GABA) transporter inhibitor NNC 711, SVS induced an increase in GABA to 150±15% of baseline in the substantia nigra (SN) of unlesioned animals, but had no effect in the pedunculopontine nucleus (PPN), the striatum or the ventromedial thalamus (VM). Dopamine release remained stable in all areas, as did GABA and amine concentrations in the SN of unstimulated controls. Following SVS, a sustained increase in GABA concentrations was observed in the ipsilesional, but not in the contralesional SN of 6-OHDA hemilesioned rats. In contrast, L-DOPA treatment produced a similar increase of GABA in the ipsi- and contra-lesional SN.

**Conclusions/Significance:**

SVS improves rod performance in a rat model of Parkinson's disease, possibly by increasing nigral GABA release in a dopamine independent way. We propose that SVS could be useful for treating symptoms of Parkinson's disease.

## Introduction

Standard Parkinson's disease treatments with dopaminergic drugs, and sometimes deep brain stimulation, often fail to alleviate axial rigidity and gait problems. Axial symptoms are strongly related to the risk of falls and better treatment options could be of great benefit to a large group of patients with Parkinson's disease [1].

Recent findings suggest that a sufficient level of noise may be necessary for normal function of the central nervous system [2], [3], [4], [5], [6]. Under some circumstances, adding noise to a system will result in improved signal detection or linearity of responses [7], [8], [9], [10]. This phenomenon can be predicted from mathematical models and is often referred to as stochastic resonance, or in a wider sense, noise benefit [11]. Several recent studies describe cross-modal noise benefit in cognitive tasks in healthy subjects [12], [13], [14], [15], [16], indicating that noise benefit is not restricted to signal detection but can be observed also when the outcome is a complex and abstract behavior. In a computational model of the degenerating brain, higher levels of external noise were needed for optimal function under conditions representing aging and loss of plasticity [4]. This could imply that the effects of neurodegeneration can be counteracted to some extent by introducing more noise in the central nervous system.

Noisy sensory signals can be transmitted to the central nervous system through any sensory pathway, but vestibular pathways have some characteristics that may be particularly useful for modulating motor deficits. Neurons in the vestibular brainstem nuclei influence axial motor functions, eye movements, autonomic cardiovascular reflexes, as well as spatial perception. Several cortical regions respond to vestibular stimulation [17] indicating widespread indirect effects. Galvanic stimulation of the vestibular system using DC current has been used for over a hundred years and is safe, but leads to disturbed posture and balance. However, by using stochastic current patterns, it is possible to activate the vestibular system without such adverse effects [18], [19], [20], [21]. Such stochastic vestibular stimulation (SVS) can improve balance in healthy subjects [22], [23]. In patients with neurodegenerative disease, like Parkinson's disease, there is some evidence that SVS improves motor functions [19], [24], autonomic reflexes and cognitive executive control [19]. Static postural sway is also reduced by SVS in Parkinson disease patients [25]. It is not known if there is a general mechanism for noise benefit in higher functions. A model involving altered dopamine release has been proposed [26], but experimental evidence to support that is lacking [27].

We used the 6-OHDA hemilesioned rat model to determine if SVS can improve motor functions in dopamine-deficient rats. Furthermore, we investigated how SVS might influence basal ganglia networks under normal and dopamine deficient conditions by measuring amino acid and dopamine release in intact and 6-OHDA hemilesioned rats.

Four nuclei that relay or mediate basal ganglia signaling were investigated in intact rats: the SN, the striatum, the PPN and the VM. Dopamine release in the striatum and SN is known to facilitate movement initiation and to occur during motor activity [28], [29], [30]. The output from the basal ganglia is inhibitory and mainly mediated by GABA-ergic neurons projecting from the internal globus pallidus (entopeduncular nucleus in rodents) and the SN *pars reticulata* to thalamus and brainstem nuclei including the PPN. The PPN is of particular interest as it responds to a large number of sensory stimuli and is involved in gait initiation [31]. Furthermore cholinergic/glutamatergic PPN neurons project to the SN and influence motor functions [32].

In line with previous clinical studies, we report improved locomotion in hemilesioned rats during SVS, but we were unable to demonstrate a change in skilled forepaw use. In unlesioned rats, SVS increased extracellular GABA concentrations only in the SN. The other investigated neurotransmitters were not significantly altered in the SN, striatum, PPN and VM. In hemilesioned animals, L-DOPA injections induced similar GABA increases in the ipsi- and contralesional SN, whereas SVS induced differential effects in the two SN, suggesting that altered nigral GABA release has a role for the behavioral effects of SVS in 6-OHDA hemilesioned rats, possibly by decreasing the inhibitory activity of the ipsilesional SN *pars reticulata* neurons.

## Results

### Effects of SVS and L-DOPA on rotarod performance in 6-OHDA and sham hemilesioned rats

Unilateral 6-OHDA lesion resulted in reduced performance on the rotarod. The total time on the rod decreased by 48% from 472±80 s before the lesion to 266±86 s after (−206±45 s, paired t-test, *p*<0.01). In contrast, the sham procedure did not significantly reduce the total time on the rod (pre-sham performance: 544±135 s, post-sham performance: 430±112 s, paired t-test *p* = 0.1004).

Because individual animals differ slightly in performance from day to day the effect of treatments were evaluated in comparison to a baseline performance obtained immediately before intervention. Treatment or sham treatment was administered in a balanced pseudo-randomized order so each animal received either sham treatment or active treatment first, and the remaining intervention in a separate trial. Baseline performances immediately before SVS and sham SVS did not differ (272±75 s and 279±77 s, respectively, paired t-test, *p* = 0.526), and this was the case also with baseline performances immediately before L-DOPA and NaCl administration (265±62 s and 244±57 s, respectively, paired t-test, *p* = 0.511).

**Figure 1:**
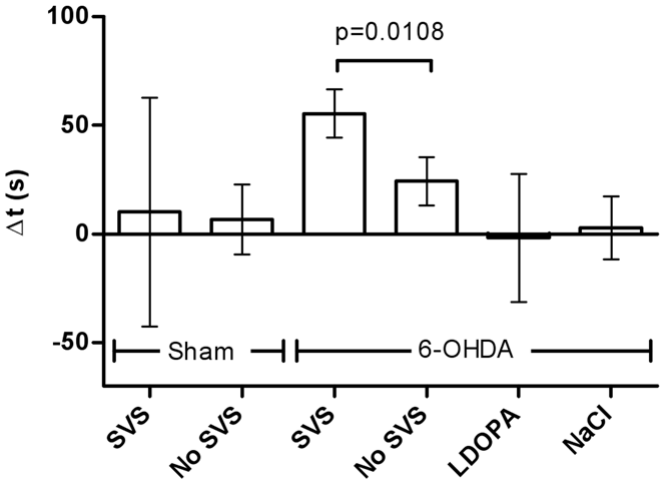
Rotarod performance after 6-OHDA or sham-hemilesions. Δt represents the change in time on rod (s, mean±SEM) compared to baseline performance on the day of the experiment. Stochastic vestibular stimulation (SVS) or no stimulation (No SVS) was administered in a counterbalanced order. In the following week a repeat experiment was performed with 6-OHDA hemilesioned animals and the effect of L-DOPA treatment or a vehicle injection (NaCl) was evaluated in counterbalanced order. P-value for paired t-test.

6-OHDA hemilesioned animals increased the time spent on rod during SVS compared to baseline significantly more than during sham SVS (Δt = 56±11 s vs. Δt = 24±11 s, paired t-test, p = 0.011, Fig. 1). The improvement observed during SVS corresponded to an increase in performance of 24±6% of the immediately preceding baseline performance. As changes in neurotransmission were sustained (see below), we also analyzed if the order of treatment had an effect. Analysis of variance with treatment and test day order as independent factors revealed no effect of test day order, *F*(1,8) = 0.0003, p = 0.987, failing to show a carry-over effect of SVS to the following test session. At group level, L-DOPA treatment did not improve the mean time spent on the rod relative to baseline and was in that respect not better than sham treatment with a saline injection (paired t-test, *p* = 0.818, Fig. 1). At the individual level, however, animals could be categorized as positive responders to L-DOPA (n = 3) or negative responders (n = 3). The positive responders increased time on rod by 60±19 s compared to the preceding baseline and this was nearly significant (p = 0.067, paired t-test) compared to saline injection (−5±7 s). In contrast, in negative responders, time on rod changed by −63±15 s compared to the preceding baseline in response to L-DOPA and by 11±30 s in response to vehicle (not significant).

Sham-lesioned animals (n = 5) did not perform better during SVS than no SVS (paired t-test for change from preceding baseline, *p* = 0.8206, n = 5. Fig. 1). Similar to L-DOPA treated hemilesioned animals the response to SVS was variable in this group. One animal improved by 192 s, and another deteriorated by 138 s during SVS. The other three retained stable performance.

### Effects of SVS on skilled forelimb use

Forelimb use was evaluated with the Montoya staircase test. As expected there was a significant decrease in the ratio of pellets picked up with the contralesional forelimb following a 6-OHDA hemilesion (0.93±0.1 prelesion vs. 0.65±0.1 postlesion, paired t-test, *p* = 0.035). No statistical difference was found between the total number of pellets retrieved before and after the 6-OHDA lesion. Unlike 6-OHDA hemilesioned animals, the sham-lesioned animals retrieved a higher number of pellets after the lesion procedure than before, suggesting continued performance improvement, and they did not develop a side preference (Table 1). For technical reasons we were unable to administer SVS during the skilled forelimb task, and we did not observe any change in the total number of retrieved pellets or the ratio retrieved with the contralesional forelimb when animals were tested immediately after a 30 minutes SVS-session (Table 1).

**Table 1:** Montoya staircase performance before and two weeks after a hemilesion procedure.

		Pre lesion	Post les	*p-value*	No SVS	SVS	*p-value*
**6-OHDA**	*Total*	51±2	45±3	*0.377*	45±3	46±3	*0.753*
(n = 5)	*Ratio*	0.93±0.1	0.65±0.1	*0.035*	0.58±0.1	0.54±0.1	*0.618*
**Sham**	*Total*	40±3	50±3	*0.005*	50±4	46±3	*0.497*
(n = 3)	*Ratio*	1.23±0.2	1.05±0.1	*0.239*	1.25±0.3	1.10±0.2	*0.520*

Total number of pellets retrieved and the contralesional/ipsilesional ratio is given (mean±SEM) Three weeks post lesion, the effect of stochastic vestibular stimulation (SVS) or no stimulation (No SVS) was evaluated on different days in a counterbalanced order. P-values for paired t-tests.

### Effects of SVS on dopamine and amino acids in un-lesioned animals

**Figure 2:**
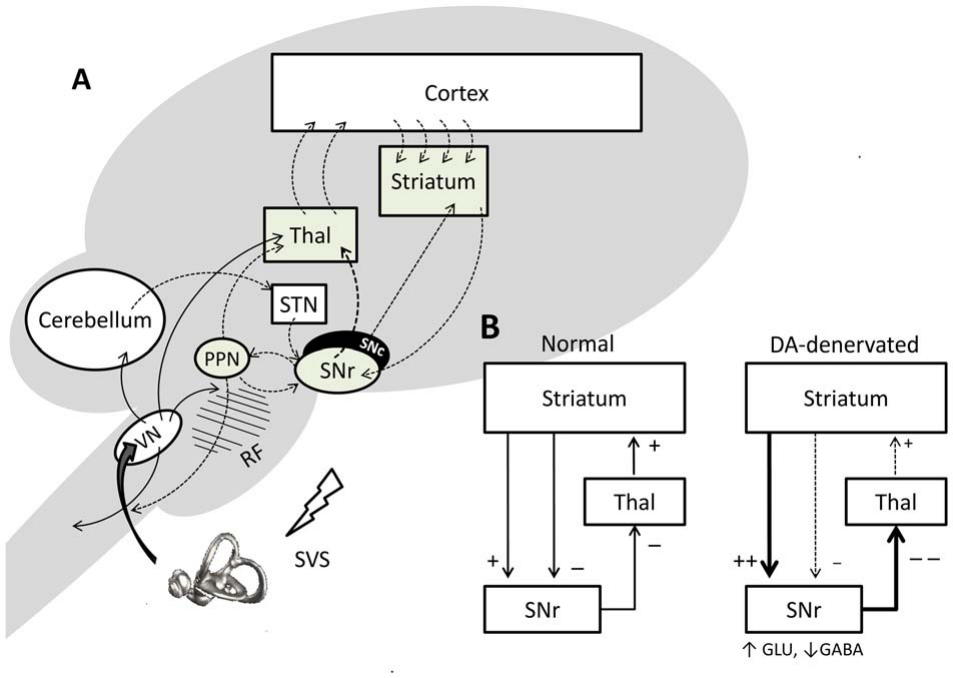
Vestibular pathways that may influence basal ganglia transmission. A) SVS is expected to activate multiple pathways from the vestibular nuclear complex (VN). Of particular interest may be pathways that connect the cerebellum and the basal ganglia over the subthalamic nucleus and thalamus [48], [49]. We chose to sample the SN, the striatum, the PPN and the VM for dopamine and amino acid concentrations before, during and for 60 minutes after stochastic vestibular stimulation in unlesioned animals. Panel B indicates the activity of the direct and indirect loop projections to the SN in normal intact rats and after nigral dopamine cell degeneration. Loss of nigrostriatal dopamine neurons lead to hyperexcitation of SN *pars reticulata* neurons, which can be counteracted by increased GABA release following L-DOPA treatment. SVS also increases nigral GABA concentrations, but the pathways involved in this effect remains to be elucidated. RF: reticular formation, SNc: Substantia nigra *pars compacta*, SNr: Substantia nigra *pars reticulata*, STN: subthalamic nucleus, SVS: stochastic vestibular stimulation, Thal: thalamus.

We used un-lesioned animals to broadly investigate if SVS induces any detectable changes in neurotransmitter release in four key brain regions in, or connected to, the basal ganglia (Fig. 2). Extracellular concentrations of dopamine, dopamine metabolites and amino acids were sampled from microdialysis probes in the SN, the striatum, the PPN and the VM before, during and after SVS. The GABA transporter inhibitor NNC 711 was retrodialysed to amplify synaptic release of GABA as we have previously demonstrated that rapid increases in GABA transmission can otherwise go undetected [33].

**Figure 3:**
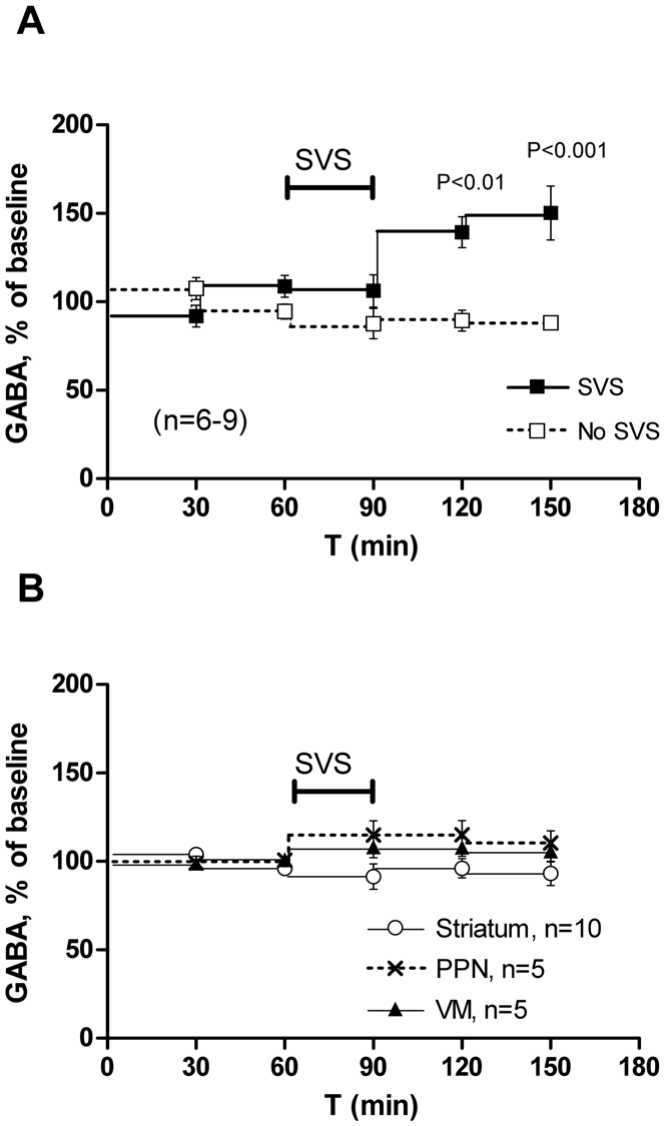
Microdialysate concentrations of GABA during stochastic vestibular stimulation (SVS) in intact animals and untreated intact controls. Percent of baseline, mean±SEM is shown. The GAT-inhibitor NNC 711 was included in the dialysates throughout the experiment. The SVS period is indicated by a horizontal bar. A. Nigral GABA concentration in SVS treated intact animals (n = 9) and untreated controls (n = 6). P-values from Bonferroni corrected post hoc tests following two-way ANOVA for timepoints T = 90–T = 150 minutes, indicating significant interaction between treatment and time F(2,26) = 3.53, p = 0.044. B. GABA concentrations in the PPN, VM and striatum of intact animals following SVS.

Following SVS in un-lesioned animals, dialysate GABA concentrations from the SN increased to 150±15% of baseline (at T = 150 min), while GABA concentrations in control animals that received no SVS were stable (Fig. 3A, Two way ANOVA, main effect of treatment F(1,26) = 14.41, p = 0.0022, time F(2,26) = 3.83, p = 0.035 and interaction treatment×time F(2,26) = 3.53, p = 0.044). The deadspace of tubings introduced a delay of approximately 20–30 minutes in the microdialysis measurements. This means that the observed increase in GABA started early during SVS-stimulation and persisted for at least 30 minutes after stimulation was terminated. DA concentrations remained stable in the SN and striatum of SVS-treated animals during SVS and the following 60 min (Fig. S1).

DA concentrations were below detection levels (0.05 nM) in the PPN and VM throughout the experiment. No significant changes in GABA concentrations were measured in the PPN, VM, or the striatum following SVS in un-lesioned animals (Fig. 3B). Concentrations of glutamate, aspartate, glycine, taurine, serine, alanine, DOPAC and HVA were not significantly affected by SVS in any of the investigated regions (not shown), but in the SN, there were large variations in glycine and glutamate concentrations after SVS (Fig. S2).

### Effects of SVS and L-DOPA treatment on dopamine and GABA in 6-OHDA hemilesioned animals

Based on our findings in unlesioned animals, dopamine and amino acid concentrations were measured in the bilateral SN of some hemilesioned 6-OHDA animals, during and after SVS and L-DOPA treatments. These animals were not tested behaviorally, but the degree of dopamine lesion was assessed after the experiment. The effects of treatments on the nigral concentrations of DA, GABA and glutamate were evaluated and compared between the ipsilesional and contralesional SN.

In the 6-OHDA hemilesioned animals there was a trend towards lower baseline GABA concentrations in the ipsilesional SN at the beginning of sampling, but the difference was not significant (ipsi_n = 6_: 24±6 nM vs. contra_n = 6_: 50±20 nM, *p* = 0.353, paired t-test. In unlesioned animals, n = 15, absolute baseline concentrations were 50±17 nM).

**Figure 4:**
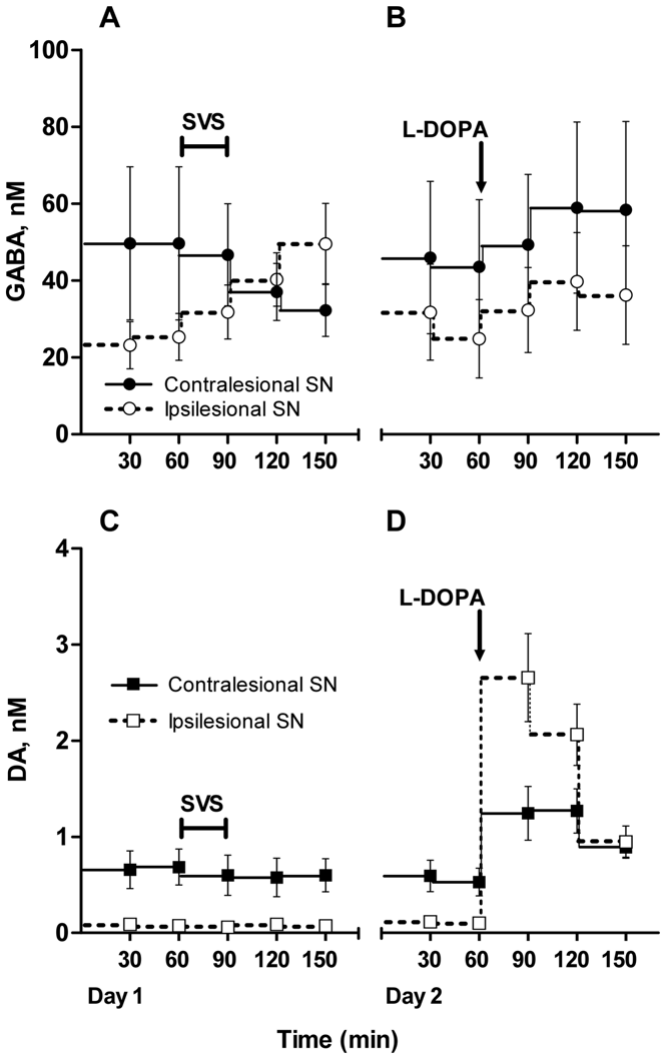
Absolute concentrations of GABA and dopamine in the ipsi- and contralesionalSN of hemilesioned 6-OHDA animals. Panel A–B shows the GABA concentrations and panel C–D the simultaneous dopamine (DA) concentrations following stochastic vestibular stimulation (SVS) and L-DOPA treatment (nM, mean±SEM). Panel A and C are measurements from day 1 and B and D from day 2. NNC 711 (30 µM) was present throughout the experiment and left in the microdialysis tube that was re-sealed over night. SVS treatment is indicated by a horizontal bar and the L-DOPA injection by an arrow.

Following SVS, the absolute GABA concentration changed differently in the two SN. In the ipsilesional SN GABA concentrations increased and in the contralesional SN they tended to fall. This differential effect is expressed as a significant interaction between time and SN side in a two way ANOVA using the absolute GABA concentrations at t = 90 to t = 150 minutes as the independent variable (Fig. 4A, F(2,20) = 4.89, *p* = 0.019).

**Figure 5:**
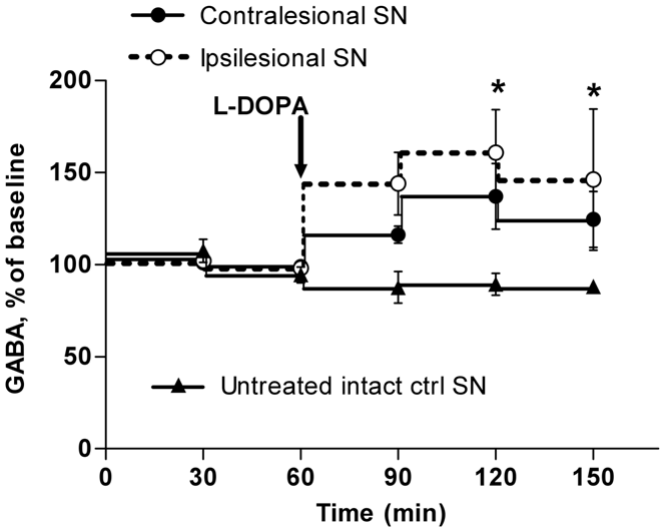
Relative changes in GABA concentrations in the ipsi- and contralesional substantia nigra of hemilesioned 6-OHDA animals following L-DOPA treatment. The microdialysate concentrations of GABA (percent of baseline, mean±SEM, n = 5–7) in the bilateral substantiae nigrae (SN) of 6-OHDA hemilesioned rats following an i.p. injection of L-DOPA are compared to GABA concentrations in the SN of untreated control animals (n = 6). Two-way repeated measure ANOVA for t = 90–t = 150 with L-DOPA treatment and time as main factors indicated a significant effect of treatment F(2,30) = 4.68, p = 0.026. * p<0.05 in post hoc Bonferroni corrected t-tests comparing ipsilesional SN in treated animals to control SN.

In contrast, L-DOPA treatment on day two produced roughly parallel changes in absolute GABA concentrations in the two SN, despite apparent asymmetries in the dopamine concentrations in the two SN following the L-DOPA injection (Fig. 4B). The relative changes in GABA concentrations in the bilateral SN following L-DOPA treatment (Fig. 5) was of similar magnitude to that observed after SVS in intact animals (Figs. 3, 5) and in the ipsilesional SN, GABA concentrations (percent of baseline) were significantly increased compared to untreated control animals at T = 120 (Fig. 5). Due to tubing deadspace, that timepoint corresponds to approximately 30–40 minutes after L-DOPA administration.

Like in unlesioned animals SVS did not alter DA concentrations in the bilateral SN of 6- OHDA hemilesioned animals (Fig. 4C). After the L-DOPA administration the DA concentrations increased in both ipsi- and contralesional SN and peaked in the first succeeding sample (T = 90, Fig. 4D). This indicates that DA levels peaked within 10 minutes after the L-DOPA injection. The increase in DA following L-DOPA treatment was in absolute terms more than two times larger in the ipsilesional SN (ipsin = 4: +2.0±0.3 nM, contra_n = 5_: +0.9±0.2 nM, p = 0.028, unpaired t-test), but peak values were not significantly higher in the ipsilesional SN (ipsin = 4: 2.3±0.4 nM, contra_n = 5_: 1.5±0.2 nM, p = 0.119). For technical reasons however, only two measurements could be obtained from the ipsilesional SN at T = 90, so peak values from the ipsilesional SN may be underestimated.

Concentrations of the amino acid glutamate in dialysates from the two SN of 6-OHDA hemilesioned animals were also analyzed. Following SVS, as well as L-DOPA injection, no significant changes or differences between the two sides were observed (Fig. S3.).

## Discussion

This is to our knowledge the first study to evaluate motor effects of stochastic vestibular stimulation (SVS) in rodents, and the first to explore the neurochemical effects of SVS *in vivo*. Stochastic resonance in central nervous system function has previously mainly been studied as a phenomenon, without trying to understand how activity patterns in the brain may change. This study demonstrates that SVS improves rod performance in hemilesioned rats. SVS was associated with increased GABA concentrations in the SN, but not with altered dopamine transmission in normal animals. In hemilesioned rats SVS increased GABA in the ipsilesional SN by a magnitude similar to that observed after L-DOPA treatment. As described previously [34], L-DOPA treatment increased nigral DA release. In contrast, SVS was not associated with altered dopamine release in lesioned animals.

For technical reasons we used two different stimulation setups and stimulation currents were not identical (Fig. S4). The current used in behavioral experiments and microdialysis experiments in hemilesioned animals contained more frequencies over 30 Hz than the current used in microdialysis in unlesioned animals. Although vestibular neurons can support activity frequencies well over 100 Hz, the vestibular system is optimized for frequencies up to ∼30 Hz [35]. The low frequency content of the signal can therefore be expected to have a higher impact on brainstem activity. The neurochemical effects of the two currents were however similar, and importantly both protocols stimulated vestibular afferents in a way that did not cause observable nystagmus or balance deficits.

### Behavioral effects of SVS

Stochastic resonance (SR) is characterized by a biphasic response curve shaped as an inverted U [5]. The design of this study did not include more than one noise level per animal, so we cannot know if the observed improvement in rod performance involves SR. A SR phenomenon has been demonstrated previously for balance improvement with SVS [22], and adding noise with small amplitude, both electrical and mechanical, to proprioceptive pathways improves balance [36], [37]. The observation that 6-OHDA hemilesioned, but not sham lesioned animals, improved rod performance can be interpreted as support for the notion that brains with impaired function display more noise benefit than the normally working brain [4], [26], [27]. The lack of improvement in sham lesioned animals is not likely to be only a ceiling effect, as the group response included both large improvement and impairment in response to SVS. Although SR can serve as a theoretical framework for understanding the effect of noisy sensory stimulation like SVS, it remains an unproved hypothesis that the effects of external noise on central nervous system functions involve SR. SVS could alternatively be viewed as a method to specifically activate the vestibular system without the adverse effects associated with stimulation with regular pattern.

SVS improved rod performance which is an integral measure of balance and locomotion. Improvements in time on an accelerating rod indicate not only improved endurance but also an increase in motor and balance functions, because the difficulty of the task increases gradually. The effect of SVS on rod performance was of similar magnitude as the effect of a single L-DOPA injection in positive responders, suggesting that the efficacy of SVS in terms of improved rod performance is not marginal. Furthermore, unlike L-DOPA treatment, SVS improved the rod performance in all lesioned animals.

The vestibular pathways are mainly influencing axial motor systems and a stronger effect of SVS on balance and locomotion than on appendicular motor control can therefore be expected. As there are reports of cross modal function improvements following noisy sensory stimulation [16], [38], we hypothesized that skilled forelimb use would also improve in response to SVS. We were, however, unable to demonstrate any effect of SVS in the Montoya stair case test. It therefore appears that noise benefit did not spread to forepaw motor function. Because SVS could not be administered in the Montoya box, an alternative explanation is that the effect of SVS wanes off rapidly. However, microdialysis measurements show that the increase in nigral GABA release is sustained for at least 30 minutes after SVS, making that explanation for the lack of observed effects on skilled forelimb use less likely. Future studies should also evaluate neurotransmission in the entopeduncular nucleus, the rodent equivalent of globus pallidus *pars interna*, an output structure which is analogue to SN *pars reticulata* and in more direct control of appendicular motor programs in humans [39].

A positive effect of SVS on axial muscle functions may have some clinical implications because axial rigidity and imbalance in Parkinson's disease are often difficult to control with L-DOPA and subthalamic DBS [40], [41].

### The effects of SVS on neurotransmission

The detection of rapid increases in GABA release with microdialysis sampling is improved by using a GABA re-uptake inhibitor (NNC 711) in the perfusion fluid [33]. One concern with this approach is that the re-uptake inhibitor may prolong and extend the increase in GABA concentrations and that this could lead to secondary changes in network activity. The increase in nigral GABA release following SVS is, however, not a long term effect of NNC 711 retrodialysis, because GABA concentrations remained stable in retrodialysed animals that did not receive SVS but were only retrodialysed with NNC 711 (Fig. 3A). Furthermore, the increase occurred selectively in the SN and not in the other investigated nuclei following the vestibular stimulation.

The microdialysis measurements did not reveal any other significant changes in neurotransmitter concentrations following SVS than the increase in nigral GABA. Because microdialysis measurements of amino acids do not necessarily reflect synaptic release activity [42], at least not without using a reuptake inhibitor or stimulated release, this does not exclude altered activity and release of neurotransmitters other than dopamine and GABA in the investigated regions. In microdialysates from the SN, mean values of glutamate and glycine increased following SVS, but the change was not significant. It may be possible to determine if this represents an increase in afferent activity by measuring stimulated release or reuptake inhibitors. Immediate early gene expression may also be informative regarding by which mechanism nigral GABA release increases following SVS.

Nigrostriatal dopamine lesions lead to increased activity of the indirect loop and decreased activity of direct loop [43], [44], and a decrease in GABA release in the SN can therefore be expected (Fig. 2). Increasing GABA release in the SN disinhibits the activation of motor programs and counteracts Parkinsonism. This is illustrated by the increase in nigral GABA concentrations that was observed following L-DOPA treatment, a treatment that is known to ameliorate Parkinsonism. There are obvious similarities between the nigral GABA increases observed after SVS in intact animals and the nigral increases in GABA after L-DOPA treatment of hemilesioned rats, not least regarding the timing of the GABA increase. Because SVS was not associated with altered dopamine release in striatum (or SN) it appears that the SVS-induced increase in nigral GABA release is not mediated by the action of dopamine on striatonigral GABA neurons. It is beyond the scope of this article to determine which pathways may mediate the observed nigral GABA increase following SVS, but as indicated in Fig. 2, vestibular neurons project to the cerebellum and the *formatio reticularis*, and possibly the PPN; all structures that may directly or indirectly alter neurotransmission in the SN. The subthalamic nucleus was not investigated in this study but may be of particular interest as DBS of the subthalamic nucleus has well documented antiparkinsonian effects, and increases nigral GABA and glutamate release [45].

Surprisingly, SVS did not induce similar changes in GABA release in the ipsi- and contralesional SN. Despite the bilateral nature of SVS the changes in GABA release in the two SN were in opposite direction, towards a more balanced GABA release between the two SN. Although we have no explanation for this phenomenon, it may have some relevance for the consistent improvement in rod performance that was observed after SVS, but not after L-DOPA treatment. Because L-DOPA increases nigral GABA release bilaterally, it may not reduce the imbalance in locomotor functions that is detrimental for rod performance. This could perhaps also explain why the initial laterality of Parkinsonism remains clearly observable throughout the course of the disease also in optimally treated Parkinson's disease patients.

### Conclusion

SVS improves rod performance in 6-OHDA hemilesioned rats. We observed a prominent and sustained increase in GABA release in the SN following SVS, but no change in dopamine release in the SN or striatum. We propose that vestibular stimulation leads to a dopamine independent disinhibition of basal ganglia output that could promote movement initiation. We suggest that it should be evaluated as a treatment alternative for Parkinson's disease, in particular with prominent axial involvement.

## Materials and Methods

### Animals

Experimental design and procedures on unlesioned animals were carried out in compliance with the UK Animal (Scientific Procedures) Act 1986 (unlesioned animals) and approved by the UK Home Office under project license no 60/3334. Experimental design and procedures for experiments involving hemilesioned and sham lesioned animals were carried out in compliance with the European Communities Council Directive of November 24th, 1986 and approved by *Göteborgs djurförsöksetiska nämnd*, the local ethics committee in Gothenburg, Sweden, under project license no. 331/10. Experiments on unlesioned animals were performed using 18 male Lister-Hooded rats (120–150 g, age 5–6 weeks, Charles River Ltd, UK), 10 of these received microdialysis probes in striatum/SN and 8 received probes in PPN/VM. Experiments with 6-OHDA and sham-lesioned animals were performed using 32 female Sprague-Dawley rats (150–200 g, age 6–8 weeks, Charles River, Germany). For the microdialysis tests 12 of these were lesioned and 4 were sham lesioned. The other 16 animals were used for behavioral tests subsequent to a hemilesion in 11 of them and a sham-hemilesion in 5 of them. Animals were housed 4–6 per cage under standard controlled environmental conditions. After implantation of microdialysis guides they were single-caged for the remainder of the experiment.

### Surgical procedures

All procedures were performed in deep surgical anesthesia (1.7–2.5% isoflurane). The surgical area was shaved and disinfected before lidocaine (1%) was infused subcutaneously for pre-emptive analgesia. Ketoprofen (5 mg/kg, s.c.) was administered as needed for post surgical analgesia.

### 6-hydroxydopamine hemilesion

The animal was placed in a stereotactic frame with bregma and lambda in the horizontal plane. After exposure of the skull, a hole was drilled over the medial forebrain bundle (4.2 mm posterior and +/− 1.2 mm lateral to bregma). A 150 µm diameter fused silica capillary was lowered 8 mm below the dura mater and 10 µg of 6-OHDA (Sigma Aldrich) dissolved in 0.9% NaCl, 0.3% ascorbate, 5 µg/µl, was injected over 2 minutes. The capillary was slowly removed after another minute and the brain surface was covered with periost membrane before closing the wound. Sham-treated animals received the saline ascorbate vehicle only.

### Implantation of vestibular electrodes

Bilateral vestibular stimulation electrodes were attached by securing the 1 mm peeled end of a teflon coated stainless steel wire (0.2 mm diameter) over the horizontal canals of the two labyrinths by pushing short wire loops through the most ventral ends of the bilateral petrosal crests. The electrode wires were fixed to an acrylic cement foundation on the skull and externalized. Animals receiving microdialysis implants were implanted with electrodes in the same surgical session.

### Microdialysis probe implantation

The unlesioned animals were stereotactically implanted with guide cannulae two days before microdialysis experiments. The skull was exposed and holes were drilled over two of the target nuclei. Targets were identified in the Paxinos and Watson rat brain atlas [46] and the coordinates relative to bregma were for the striatum: +1.0 mm anterior, 2.8 mm lateral, for the SN: 5.3 mm posterior, 2.1 mm contralateral, for the PPN: 8.0 posterior, 1.8 mm lateral, and for the VM: 2.6 posterior, 1.5 mm contralateral). Holes were also drilled in the parietal bones to fasten two stainless steel jewellers' screws. The dura was opened and the underlying pial membrane was opened by pulling it gently with fine forceps. Microdialysis guides (MAB 4.15.IC, Microbiotech, Sweden) were slowly implanted to a depth immediately above the target nucleus (6.0 mm from the brain surface for the PPN and VM, 3.2 mm and 6.2 mm for the striatum and the SN, respectively). The guide cannulae were secured to the skull with acrylic dental cement.

Some 6-OHDA hemilesioned animals were chronically implanted with in-house constructed microdialysis probes positioned bilaterally in the SN as described previously [47] and provided with vestibular electrodes as described above. Animals were allowed to recover for 24–48 h before the first microdialysis experiment.

### Microdialysis

Two days after implanting the microdialysis guide cannulae, the unlesioned animals were briefly anaesthetized to insert bilateral microdialysis probes (Cuprophane membranes extending 1 mm, or with striatum guides, 3 mm, below the guide, MAB 4.15, Microbiotech, Sweden). The probes were continuously perfused with a Ringer solution containing 140 mM NaCl, 3.0 mM KCl, 1.2 mM CaCl2 and 1.0 mM MgCl2 and a GABA reuptake inhibitor, NNC 711 (30 µM, Tocris Cookson Ltd, UK). The animals were allowed to recover and were then kept in a standard microdialysis bowl, with the fluid lines from the microdialysis probes connected to the external equipment via a swiveled balance arm. The perfusion rate was kept at 4.0 µl/min for one hour and was then lowered to 1.5 µl/min. Baseline sampling commenced after 2 hours of perfusion. Microdialysis samples were collected every 30 minutes. After two baseline samples, stochastic vestibular stimulation commenced. Microdialysis perfusion continued throughout the 30 minute stimulation period, and for another 60 minutes, providing a total of 5 microdialysis samples from each probe. The samples were split and analyzed for amino acid content as described previously [33], and for amine content by two-dimensional HPLC with electrochemical detection as described by Lindgren et al. [34].

The 6-OHDA hemilesioned animals were subjected to a repeat microdialysis experiment on the following day. The experiment protocol was as described above, but instead of a 30 minutes stimulation period, they were injected with L-DOPA and benserazide (6 mg/kg and 12 mg/kg, respectively, i.p.) after the second sampling period.

After the last microdialysis experiment animals were euthanized and the brain was removed and fixed in paraformaldehyde. The brains were sectioned and probe locations were determined with reference to the Paxinos and Watson rat brain atlas [46]. Results from probes with less than 50% of the active membrane in the target nucleus were excluded. This reduced the final n of investigated brain areas to 5 for PPN and VM and 10 for striatum, 9 for SN respectively in intact animals. In 6-OHDA lesioned animals, a total of 6 probes were included in the lesioned SN, and 6 in the unlesioned. The positions of the active membranes of all included probes are indicated in Fig. S5.

### Stimulation protocol

Current was delivered through the bilateral electrodes using two setups. Unlesioned animals were stimulated using two stimulus isolators (NeuroLog NL800, Digitimer Ltd. Hertfordshire, UK) connected to a biphasic pulse buffer unit (NeuroLog NL512) and a stimulus pattern fed from a voltage source pulse generator or a DAT recorder with a prerecorded stochastic stimulation (<30 Hz) pattern. For lesioned animals a bipolar analogue stimulus isolator (model 2200 A-M Systems, Sequim, Washington, U.S.A) was used, and the stimulus pattern was fed from a low pass (<30 Hz) filtered voltage source generator (BitScope 100 with BitGen coprocessor, Metachip Pty. Ltd., Australia), which was programmed to generate sinusoid or white noise patterned voltages.

Bipolar 1 Hz sinusoid stimulation pattern was used to determine an appropriate maximum current. The amplitude was slowly increased until a 1 Hz rocking of the animals head was clearly visible. The lowest amplitude A that reproducibly produced head rocking was used as maximum amplitude during the stochastic vestibular stimulation period of the experiment.

The mean maximum stimulation amplitudes are given in Table 2, and were typically between 0.2 and 0.6 mA. The stochastic stimulation protocol consisted of a 30 minutes sequence containing bipolar current with random amplitude between (−A) and (+A) and random frequencies <30 Hz. Examples of the stimulation currents are given in Fig. S4.

**Table 2:** Maximum stimulation amplitudes (mA, mean±SEM) in the different experiments.

Experiment	Animals	Mean±SEM (mA)
Rotarod	6-OHDA hemiles (n = 6)	0.30±0.02
	Sham hemiles (n = 5)	0.27±0.03
Montoya staircase	6-OHDA hemiles (n = 5)	0.23±0.05
	Sham hemiles (n = 3)	0.27±0.03
Microdialysis	SN/ST, unlesioned (n = 9)	0.48±0.02
	PPN/VM, unlesioned (n = 5)	0.41±0.03
	Bilat SN, 6-OHDA hemiles (n = 7)	0.47±0.08

Stimulation amplitudes were determined by subjecting the animals to a sinusoid current that was increased until head rocking was just visible. During stochastic stimulation the amplitude never exceeded ± this amplitude. A different current delivery system was used for the microdialysis experiments in unlesioned animals. 6-OHDA 6-hydroxy-dopamine, PPN pedunculopontine nucleus, ST striatum, SN substanta nigra, VM ventromedial thalamus.

### Motor behavior – Rotarod

Animals were trained to run on a 6 cm (ø) accelerating rod. Training and a set of prelesion baseline tests took place before any surgical procedure, and animals were re-tested three weeks after sham/6-OHDA lesion procedures. Each baseline test and experimental test episode consisted of four 10 minute long accelerations (5–40 rpm). The mean time on rod during each test episode was determined as previously described [28]. Three to five days after implantation of bilateral vestibular electrodes the animals were baseline tested and after 30 minutes tested again with or without vestibular stimulation.

**Figure 6:**
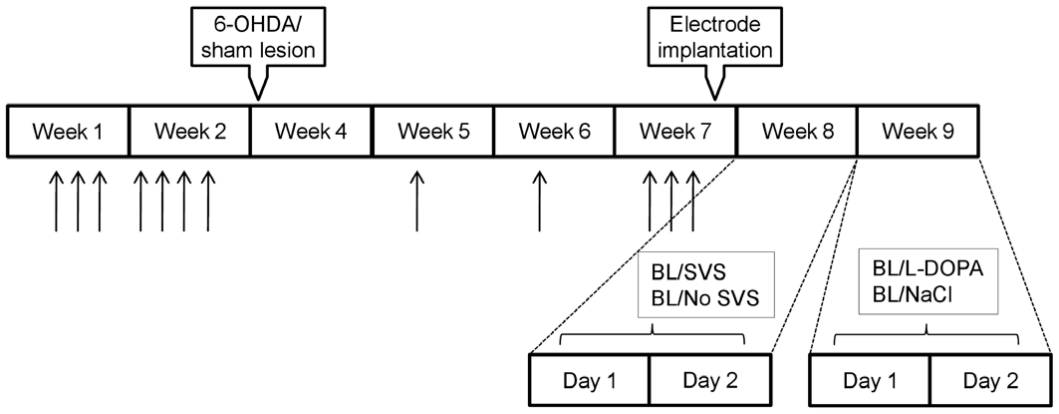
Schematic illustration of the experimental design for the Rotarod testing procedure. The black arrows represent training episodes for each animal. Rod performance during stochastic vestibular stimulation (SVS) or no SVS was compared to the baseline measurement (BL, 4×10 min before either condition) on the day of the experiment. The stimulated condition (30 min SVS in cage followed by 4×10 min testing during SVS) or non stimulated condition (30 min rest in cage followed by 4×10 min testing during no SVS) was carried out in a counterbalanced order after the BL measurements. L-DOPA testing was carried out in the beginning of the following week in counterbalanced order; BL testing followed by either an L-DOPA or NaCl injection, rest in cage for 30 min followed by testing for 4×10 min.

Stimulation was started 30 minutes before the first of four rod session, and continued throughout testing. This procedure was repeated for two days in a counterbalanced order so that each animal was tested one day with and one day without stimulation. The same procedure was carried out over an additional two day period, this time with either an L-DOPA and benserazide intraperitoneal injection (6 mg/kg and 12 mg/kg respectively) or a sham injection (NaCl 0,9%) given in a counterbalanced order (Fig. 6). The change in performance invoked by the SVS or no SVS condition was calculated with reference to the baseline performance on the same day. 6-OHDA-lesioned animals with less than 70% dopamine depletion (n = 5), as revealed by port-mortem analysis, were excluded from the Rotarod data analysis.

### Motor behavior - Montoya staircase test

Two weeks before the hemilesion procedure, rats were food restrained to 90–95% of free feeding weight and trained to retrieve sugar pellets (45 mg each; BioServ, Frenchtown, NJ, U.S.A.) in a Montoya staircase apparatus (9×6×30 cm, Campden Instruments Ltd, Loughborough, UK). Training took place between 9am–1pm, over a period of 5 days and consisted of 2×15 min sessions in the box with 1 h 45 min rest between sessions. The box was covered with a dark cloth to maintain a dark and constant environment throughout training and testing. Animals which after initial training retrieved at least 9 pellets per side were included in the experiment. Seven days after implantation of vestibular electrodes the rats were food restrained and baseline tested. They were tested again over the following two days with and without vestibular stimulation in a counterbalanced order. Stimulation was applied for 30 minutes immediately before the animal was transferred to the test box. 6-OHDA lesioned animals with less than 70% dopamine depletion in the post mortem analysis were excluded from analysis.

### Statistical analysis

When appropriate, microdialysate concentrations were transformed to percent of baseline to reduce the effects of recovery variations. The treatment effect on neurotransmitter concentrations was evaluated by repeated measure two-way ANOVA with treatment and time as independent factors or with one sample t-tests with 100% as the theoretical mean, as appropriate. Locomotion effects were evaluated with paired t-tests comparing the change in time (s) on rod observed during stimulation or no-stimulation conditions. On the Montoya staircase, the overall number of sugar pellets consumed, total number of pellets on each side as well as the ratio between number of pellets eaten from impaired (contralesional) side and non-impaired (ipsilesional) side after vestibular stimulation or after no stimulation, were evaluated by paired t-tests. Data are given as mean±SEM, and p≤0.05 was considered statistically significant.

## Supporting Information

Figure S1 **Dopamine concentrations in dialysates from the substantia nigra and striatum of normal animals.** Figure shows dopamine (DA) concentrations (percent of baseline, mean±SEM) in dialysates from the SN and the contralateral striatum of 9 un-lesioned animals subjected to stochastic vestibular stimulation for 30 minutes (horizontal bar). Concentrations remained stable throughout the stimulation period and the following hour. For clarity, measurements from untreated control animals (n = 6) are omitted from the figure. NNC 711 (30 mM) was present in the perfusate throughout the experiment.
(TIF)

Figure S2 **Amino acid concentrations in dialysates from the substantia nigra of normal animals.** Panel A shows the relative concentrations (percent of baseline, mean±SEM) of glutamate and glycine from the SN of un-lesioned animals subjected to stochastic vestibular stimulation (SVS, horizontal bar, n = 9) or no SVS (ctrl, n = 6) for 30 minutes. Over this time there were no significant difference in relative concentrations (Two-way repeated measure ANOVA for t = 90–t = 150 with SVS treatment and time as main factors, Glutamate: F_treat_(1, 24) = 2.76, p = 0.12, F_time_(2, 24) = 0.5, p = 0.61, F_interact_(2,24) = 1.01, p = 0.38, Glycine F_treat_(1, 26) = 1.36, p = 0.26, F_time_(2, 26) = 1.63, p = 0.21, F_interact_(2,26) = 1.34, p = 0.28). The higher mean values and large variability in samples t = 120 and t = 150 coincided with increased exploratory behavior in the cage. Taurine and glutamine levels remained stable following SVS (Panel B, two-way repeated measure ANOVA for t = 90–t = 150 with SVS treatment and time as main factors, Taurine: F_treat_(1, 26) = 1.88, p = 0.19, F_time_(2, 26) = 1.63, p = 0.22, _interact_(2,26) = 0.44, p = 0.65, Glutamine F_treat_(1, 26) = 1.71, p = 0.21, F_time_(2, 26) = 1.81, p = 0.18, F_interact_(2,26) = 0.30, p = 0.75). NNC 711 (30 mM) was present in the perfusate throughout the experiment.
(TIF)

Figure S3 **Glutamate concentrations in dialysates from the bilateral substantiae nigrae of 6-OHDA hemilesioned rats.** Figure shows glutamate (GLU) concentrations (percent of baseline, mean±SEM) in dialysates from the ipsi- and contralesional SN before, during and after stochastic vestibular stimulation (SVS, horizontal bar, 30 minutes) in panel A and in response to L-DOPA treatment (6 mg/kg, i.p., arrow) in panel B. NNC 711 (30 mM) was present in the perfusate throughout the experiment.
(TIF)

Figure S4 **Stochastic stimulation current patterns.** Panel A shows the stimulation current used in normal animals and panel B the current used in hemilesioned rats. The time base is 200 ms per division, and the upmost part of each panel shows the frequency distribution.
(TIF)

Figure S5 **Placement of microdialysis probes in intact and hemilesioned animals.** Unlesioned animals are shown schematically in panel A (striatum and substantia nigra) and panel B (ventromedial thalamus and the pedunculopontine nucleus). Panel C shows the bilateral nigral locations in hemilesioned animals. Solid lines indicate the estimated location of the active dialysis membrane.
(TIF)
