# Supplementary figures and images for: Noisy Galvanic Vestibular Stimulation Promotes GABA Release in the Substantia Nigra and Improves Locomotion in Hemiparkinsonian Rats
Source: PLoS One. 2012 Jan 6;7(1):e29308. doi: 10.1371/journal.pone.0029308 (PMC3253081; doi:10.1371/journal.pone.0029308)

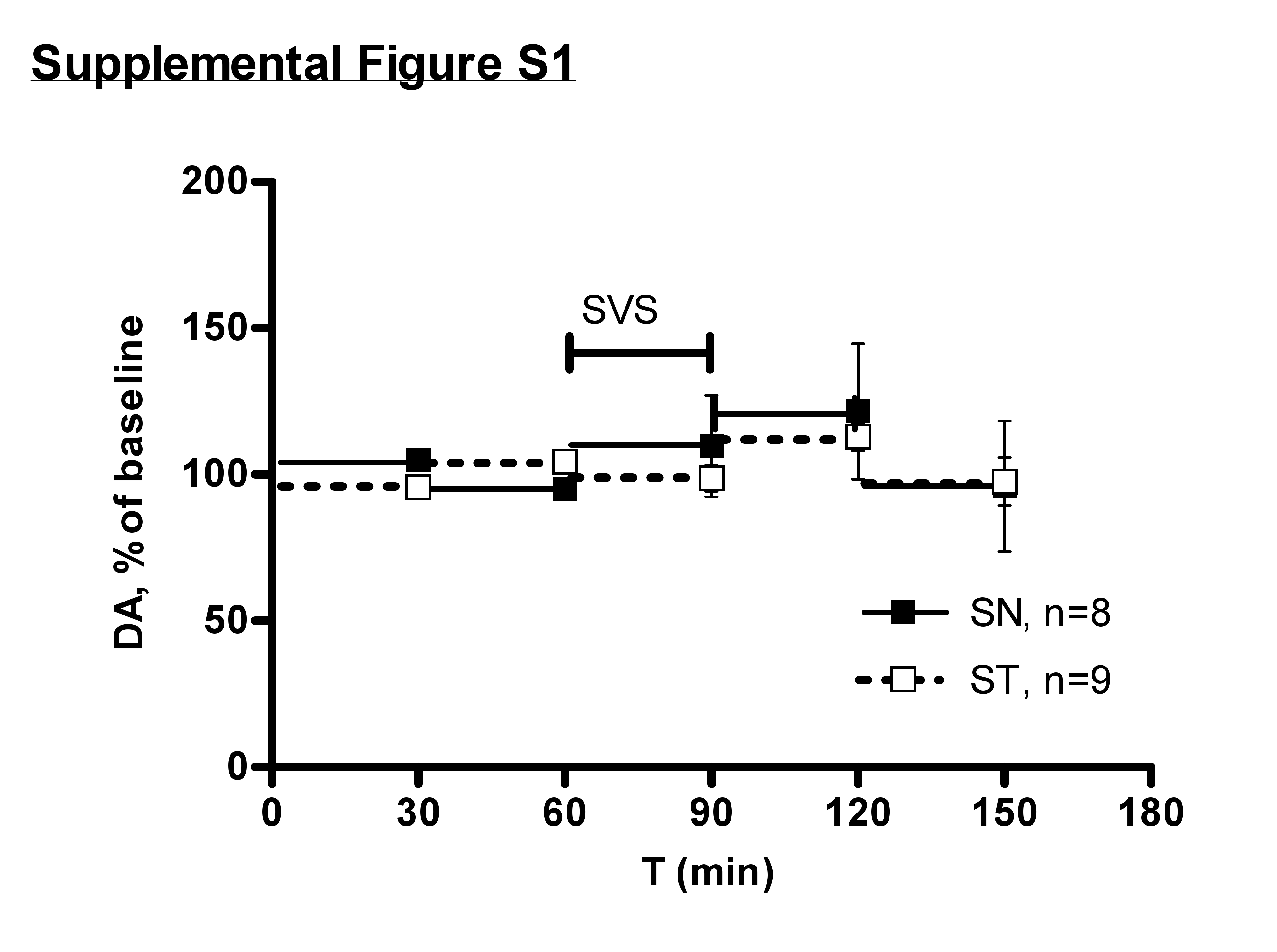

Supplement: Figure S1 — Dopamine concentrations in dialysates from the substantia nigra and striatum of normal animals. Figure shows dopamine (DA) concentrations (percent of baseline, mean±SEM) in dialysates from the SN and the contralateral striatum of 9 un-lesioned animals subjected to stochastic vestibular stimulation for 30 minutes (horizontal bar). Concentrations remained stable throughout the stimulation period and the following hour. For clarity, measurements from untreated control animals (n = 6) are omitted from the figure. NNC 711 (30 mM) was present in the perfusate throughout the experiment. (TIF) [file pone.0029308.s001.tif]

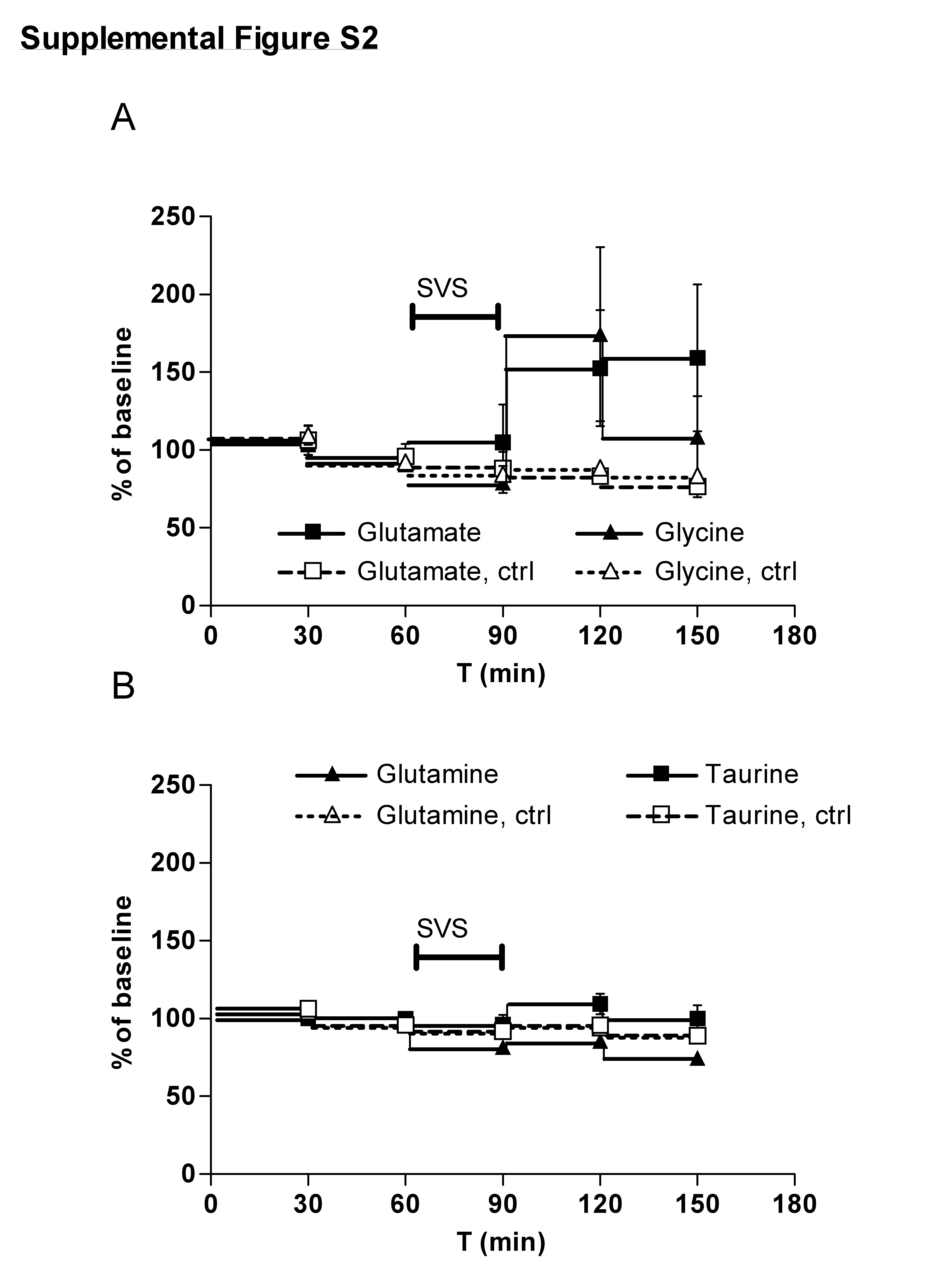

Supplement: Figure S2 — Amino acid concentrations in dialysates from the substantia nigra of normal animals. Panel A shows the relative concentrations (percent of baseline, mean±SEM) of glutamate and glycine from the SN of un-lesioned animals subjected to stochastic vestibular stimulation (SVS, horizontal bar, n = 9) or no SVS (ctrl, n = 6) for 30 minutes. Over this time there were no significant difference in relative concentrations (Two-way repeated measure ANOVA for t = 90–t = 150 with SVS treatment and time as main factors, Glutamate: Ftreat(1, 24) = 2.76, p = 0.12, Ftime(2, 24) = 0.5, p = 0.61, Finteract(2,24) = 1.01, p = 0.38, Glycine Ftreat(1, 26) = 1.36, p = 0.26, Ftime(2, 26) = 1.63, p = 0.21, Finteract(2,26) = 1.34, p = 0.28). The higher mean values and large variability in samples t = 120 and t = 150 coincided with increased exploratory behavior in the cage. Taurine and glutamine levels remained stable following SVS (Panel B, two-way repeated measure ANOVA for t = 90–t = 150 with SVS treatment and time as main factors, Taurine: Ftreat(1, 26) = 1.88, p = 0.19, Ftime(2, 26) = 1.63, p = 0.22, interact(2,26) = 0.44, p = 0.65, Glutamine Ftreat(1, 26) = 1.71, p = 0.21, Ftime(2, 26) = 1.81, p = 0.18, Finteract(2,26) = 0.30, p = 0.75). NNC 711 (30 mM) was present in the perfusate throughout the experiment. (TIF) [file pone.0029308.s002.tif]

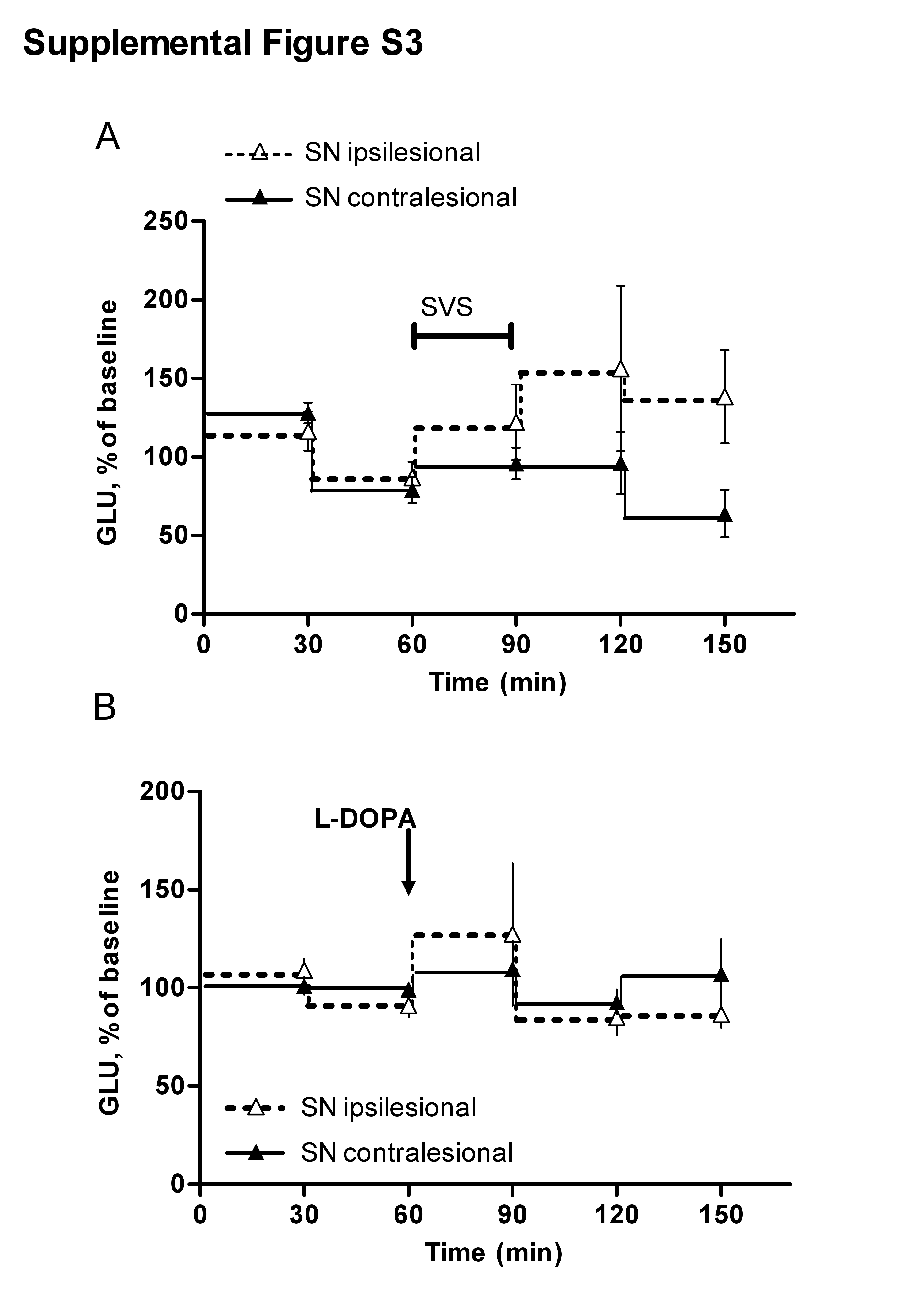

Supplement: Figure S3 — Glutamate concentrations in dialysates from the bilateral substantiae nigrae of 6-OHDA hemilesioned rats. Figure shows glutamate (GLU) concentrations (percent of baseline, mean±SEM) in dialysates from the ipsi- and contralesional SN before, during and after stochastic vestibular stimulation (SVS, horizontal bar, 30 minutes) in panel A and in response to L-DOPA treatment (6 mg/kg, i.p., arrow) in panel B. NNC 711 (30 mM) was present in the perfusate throughout the experiment. (TIF) [file pone.0029308.s003.tif]

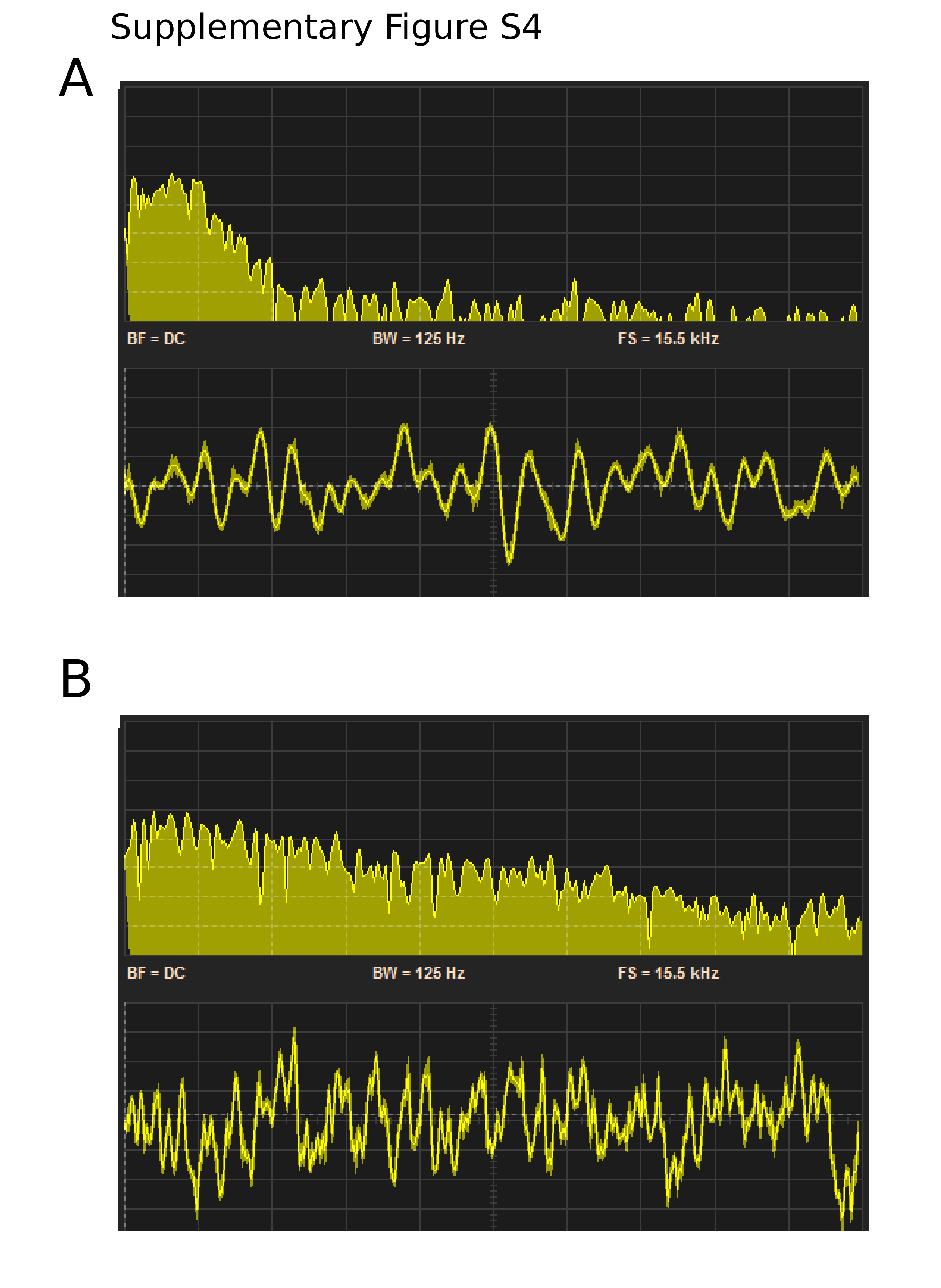

Supplement: Figure S4 — Stochastic stimulation current patterns. Panel A shows the stimulation current used in normal animals and panel B the current used in hemilesioned rats. The time base is 200 ms per division, and the upmost part of each panel shows the frequency distribution. (TIF) [file pone.0029308.s004.tif]

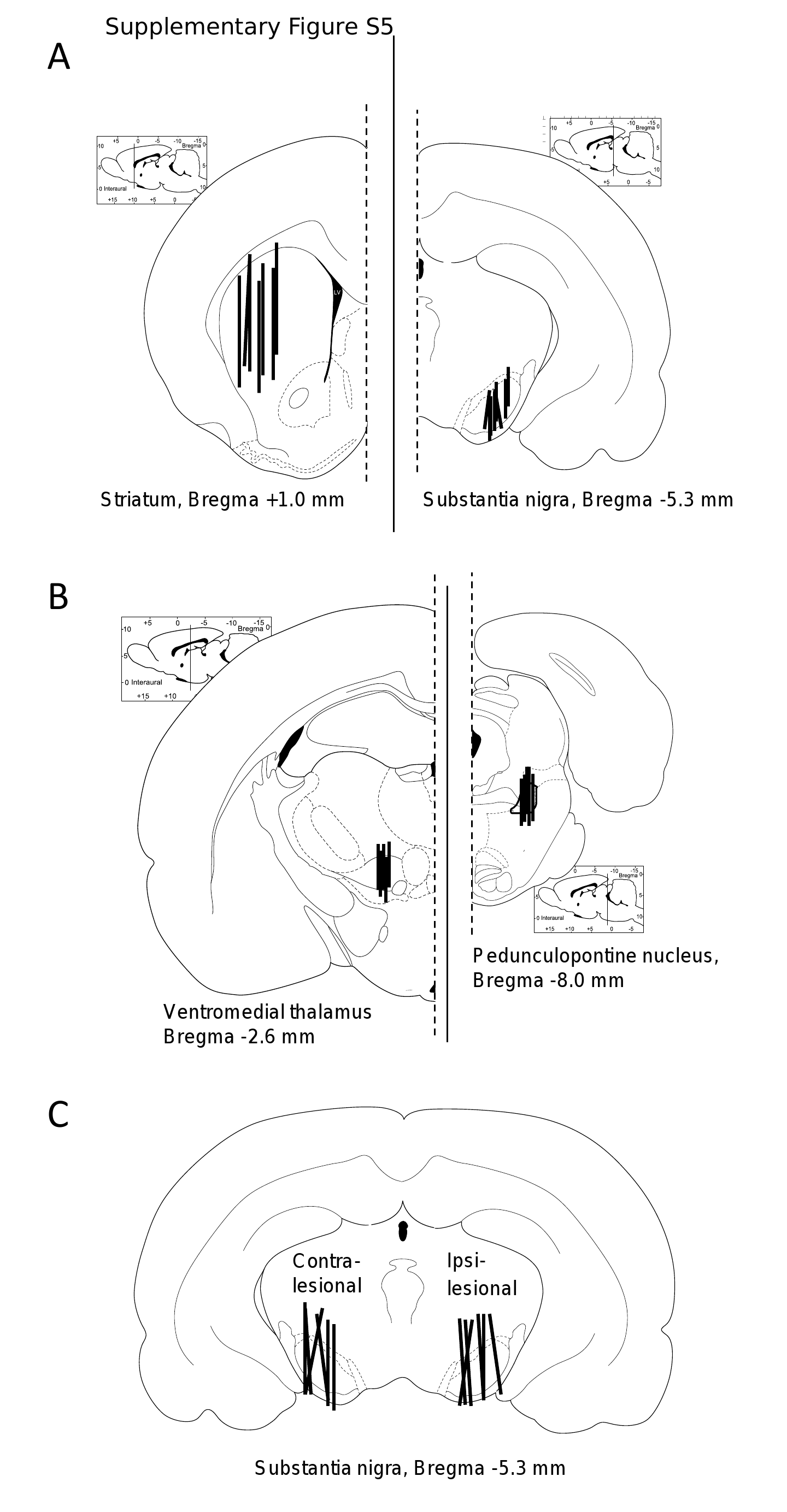

Supplement: Figure S5 — Placement of microdialysis probes in intact and hemilesioned animals. Unlesioned animals are shown schematically in panel A (striatum and substantia nigra) and panel B (ventromedial thalamus and the pedunculopontine nucleus). Panel C shows the bilateral nigral locations in hemilesioned animals. Solid lines indicate the estimated location of the active dialysis membrane. (TIF) [file pone.0029308.s005.tif]
